# Leadership Development in Medicine: A Review

**DOI:** 10.7759/cureus.65028

**Published:** 2024-07-21

**Authors:** Lulu Alwazzan

**Affiliations:** 1 Medical Education, Imam Mohammad Ibn Saud Islamic University, Riyadh, SAU

**Keywords:** leader development, medicine, social capital, human capital, management, leadership development, leadership

## Abstract

The complex and unique challenges of healthcare require physicians who are competent leaders. Leadership is shaped by complex dynamic processes and various factors. Such factors include intrapersonal (leadership identity, cognitive abilities, and self-leadership) and interpersonal factors (vision and mission development and team building). The purpose of this paper is to review current thinking and advancements in leader and leader development by drawing on the administrative literature and discussing how it may apply to medicine, focusing on the contemporary approaches, challenges, and opportunities in this field. More specifically, this review analyses the intrapersonal and interpersonal development of medical leadership, beginning with a consideration of the current state of medical education and practice. It also covers the current challenges affecting medical leadership, as well as the implications for practice, policy, and research. In this paper, it was found that leader and leadership development are both essential for leader growth and to enhance leaders' competencies and effectiveness. The latter leads to improved organizational performance. The paper also highlights the importance of integrating various methods of teaching and learning to optimize leadership development programs.

## Introduction and background

Leader and leadership development in healthcare are essential and active areas of study [1]. This scholarly interest is a response to the dire need for competent leaders and effective leadership to deal with the ever-evolving medical sector. Population growth, the emergence of new diseases and disease complications, and technological advances are among the factors that have led to the emergence of complex organizational challenges [2]. To face such challenges, leaders require innovative approaches, dynamic problem-solving skills, and a tolerance for organizational complexity. Drawing on the administration literature, the purpose of this paper is to present current thinking and advancements in leader and leadership development, focusing on the challenges, and opportunities in this field and as it relates to medicine. While the conceptual advancements offered in this literature review are not novel, they are not easily implemented. The findings of this review should inspire medical leaders to think globally, creatively address challenges, and innovate.

There is wide recognition that leadership competencies can be cultivated and developed amongst individuals and teams through intentional education and development [3,4]. Consequently, academic and medical institutions have dedicated enormous efforts and resources to provide leadership development opportunities and initiatives to cultivate the current and next generation of leaders in the healthcare sector. A distinction between the development of the leader and leadership development must be made. The former is concerned with arming individuals with the necessary competencies to lead, while the latter is a process of development that involves multiple people [5].

A "leader" is an individual who guides activities and obligations in healthcare settings such as managing, leading, and making decisions. Medical leaders often hold positions of authority, such as administrators or department heads, and are responsible for organizing and leading medical teams and systems [6]. Leadership development in medical education and practice refers to the deliberate pursuit of enhancing an individual's knowledge, talents, and competencies to effectively lead and manage in the healthcare business. It consists of mentorship, experience learning, and formal and informal training courses aimed to prepare healthcare professionals for positions of leadership [7,8]. For leader development formal leadership training programs typically include a combination of formal and informal training opportunities. Formal opportunities include didactic teaching, short courses, and experiential learning [9]. While informal opportunities include mentorship, coaching, and reflective practice [10]. By providing individuals with deliberate and structured opportunities to enhance their leadership knowledge, skills, and attitudes, institutions aim to arm a cadre of elite individuals that, as a result of their newly found knowledge and skills, can positively influence human resource development, workplace culture, and healthcare outcomes [11].

More recent approaches, however, target leadership development, that is, targeting the process of leadership that involves several people in the workplace context [12-14]. This calls for leaving behind the constant need to link leadership development to individualistic leadership theory, arguing that leadership development is a complex set of processes that occur in the context of ongoing adult learning [15]. Leadership development is multilevel and longitudinal in nature, meaning there needs to be a focus on the intra-personal and inter-personal aspects of development simultaneously and over time [16,17]. Intra-personal factors include self-leadership and formalized training programs, while interpersonal aspects include relationship-building and influencing others. 

It is critical to realize that the path to medical leadership development is challenging. The healthcare industry's often complex and dynamic structure, combined with a plethora of competing demands and limited resources, presents substantial barriers to the development of leaders and leadership in the healthcare sector [18,19]. Complexity is defined by Cohen et al. (2013) [20]: “a dynamic and constantly emerging set of processes and objects that not only interact with each other but come to be defined by those interactions”. Such processes are characterized by multiplicity (the number of elements), interdependence (the degree of connection between the elements), and heterogeneity. While often starting with the same elements, these properties often lead to unpredictable outcomes [21,22]. This makes the role of the leader more essential in leading in and through unpredictability. It is important to note that not everything in medical practice is considered complex, as some processes are complicated and outcomes can be predicted and managed.

Recent data from the literature underscore the increasing demands placed on medical leadership and the increasing importance of talented leaders in navigating the shifting healthcare landscape [1]. Additionally, the traditional hierarchical structure of medical education and practice may present barriers to implementing and sustaining leadership development initiatives [23]. Addressing these challenges requires a comprehensive understanding of the unique context and dynamics of medical practice, as well as innovative and evidence-based approaches to leadership development. By offering an understanding of leadership development in medicine, the medical sector may offer better leadership that leads to the optimization of organizational performance and enhances healthcare outcomes.

The review has not identified and discussed all the articles on leadership development, rather, it aims to present a prevalent discourse within administrative literature and to present and discuss high-level concepts within the context of current published works. The ultimate purpose of this review is to assist in the development of competent and diverse medical leaders who are capable of successfully navigating the complex and ever-changing healthcare environment in order to improve patient outcomes and the system as a whole. In what follows, this review paper elaborates on the context of medicine and presents the intra-personal and inter-personal development of leadership. Moreover, it discusses the challenges to leader and leadership development and the implications for policy, practice, and research.

Leadership in medical education and practice

The leadership training of physicians has often been categorized according to stages: undergraduate, postgraduate, and continuous education. The literature argues for integrating leadership training in undergraduate [17] and postgraduate medical curricula [4]. The most commonly cited reason for adding leadership training in medical curricula is that it will better prepare medical practitioners to manage and lead in a complex and changing healthcare environment, increasing organizational performance and patient care. Such works report formal leadership training programs. Often such educational interventions lack grounding in conceptual leadership frameworks, neglect to evaluate outcomes properly, and most focus primarily on cognitive leadership domains. In terms of training targeting more practicing doctors [1], Steinert et al. (2012) reviewed faculty development initiatives that target leadership competencies amongst medical faculty members, and they identified key features of successful programs, including the use of multiple instructional methods, experiential learning, and peer support and mentorship. Research has also taken a deeper look, advocating for the development of leadership amongst course coordinators and recommending a complex adaptive style of leadership based on whole systems theory to enhance undergraduate programs [22].

Beyond embedding leadership development in learning outcomes and educational frameworks within the work context, there is also an increasing recognition of clinical leadership, that is, leadership development rooted in work-based activities [17,23-25]. This trend shows a shift towards integrating leadership competencies directly into clinical practice, acknowledging the intrinsic role leadership plays in the clinical context. This shows that leadership is perhaps honed and developed in real-world scenarios and practical applications and that medical or clinical leadership is a distinct style of leadership. Moreover, medical leadership often involves cross-disciplinary collaboration. As a result, a common challenge is facilitating effective communication and collaboration among professionals with diverse professions and experiences [26,27]. Furthermore, navigating the complex healthcare system poses a challenge to leaders as well. Navigating complex, often conflicting, regulations and operational intricacies requires that medical leaders make strategic decisions while ensuring high quality patient care.

## Review

Individual-level leadership

According to Day et al. (2014) [5], three aspects of leader development are broadly addressed in the literature: identity development, cognitive abilities, and self-leadership. First, leader identity development in healthcare is a complex dynamic process that is shaped by various factors. Such factors include personal experiences, interpersonal relationships, formal leadership roles, and organizational culture [11]. The leadership literature describes several models for leadership identity development, one of the most well-known being the Komives et al. (2006) Leadership Identity Development Model [28]. The model categorized six stages of leadership identity development: 1) Awareness (individuals begin to recognize their potential leadership), 2) Effortful Identification (individuals begin to explore their leadership interests and abilities, 3) Involvement (take on leadership roles and responsibilities), 4) Leadership for Self-Growth (focus on developing their leadership skills and knowledge), 5) Leadership for Impact (focus on using their leadership to make a difference in their context), 6) Authentic Leadership (develop a clear sense of their own leadership identity and are able to lead).

The leadership identity development process is not linear, rather, it is complex. Within this model, individuals move back and forth between stages as they learn and grow. The model offers a conceptualization of the different stages of leadership identity development. By understanding the different stages of leadership identity development and the factors that can influence this process, individuals can better prepare themselves for leadership roles. These different stages can be achieved by explicit training and development efforts and involvement in leadership roles. In identity development, it is important to draw focus to influence others. Influence is one’s ability to motivate and mobilize followers to participate in change (steps 5 and 6 of Komive’s development model). While influence is a relational process it may be considered as an individual competency to be developed [29]. This can be appreciated through a shared social identity, once a group of people have a shared organizational identity, they are more likely to follow those who share this identity. For the leader, it is important to recognize this social identity and have the ability to influence the actions of others through it.

Another important aspect addressed in the literature is the cognitive domains vital to leader development. Such critical skills include problem-solving, planning and implementation, solution construction and evaluation, social intelligence, and self-awareness [30]. These abilities have been the locus of many leadership development programs, the premise being the more skilled the individual the more likely they are able to lead [8]. As vital as these skills are, the more senior a leader becomes in organizational positions, the acquisition of strategic and business skills becomes more important for effective performance [31].

Self-leadership is also of crucial importance at the individual developmental level; that is, being a self-directed lifelong learner [30]. Ensuring leaders are self-aware and able to diagnose themselves in terms of their learning is crucial for organizations that are continuously evolving. This is true for any organization but especially true for healthcare organizations where administrative practice is often a reflection of clinical practice and updated clinical guidelines and ways of practice rapidly evolve. Additionally, self-directed learning is an efficient and often cost-effective way to develop human capital [30], as learners self-diagnose and take charge of their own learning needs and requirements based on their current leadership assignment. 

Group-level leadership

According to Day et al's (2014) categorization, leadership development is an interpersonal process that involves a team of people to occur [5]. As a dynamic process that involves several individuals, the examination of leadership development requires a multi-level approach [29]. A simultaneous examination of the individual (their emergence and enactment of leadership, as explained in the above section), their interaction with others, and their interaction with the broader culture [31]. Here the exchange between individuals in an organization becomes central [32,33]. This exchange may be between leaders or leaders and followers. Such interactions shape social capital and human resource development. As a result, positive values have been the focus of previous studies [12]. Moreover, certain psychological processes such as knowledge, interpersonal skills, and communication competence coupled with organizational culture and environment leverage the development of strong, fruitful professional relationships, that one can draw on to accomplish organizational objectives [34,35].

Here it is important to draw a distinction between social capital and human capital. Social capital is concerned with interaction in the social context. However, human capital is concerned with the individual leader attributes [34]. Social capital is characterized by contact (e.g., mentoring), assimilation (e.g., job assignment), and identification (e.g. leadership training). These educational practices vary in their potential for social capital development; thus design of educational interventions should be done accordingly [34].

When considering leadership as a process, the literature presents the importance of social networks and the time it takes to develop such relationships. According to Cullen-Lester et al. (2016), groups of leaders must understand the leverage and the influence of their positions within an organization [30]. Such leverage can enhance a group's ability to accomplish organizational goals and enhance alignment with the overall aims of the organization.

Challenges facing leadership development

Leader and leadership development in medicine face certain challenges. First is the intra-personal development, that is the development of self-identity prior to the development of leadership competencies. It is often difficult to recognize talent, making talent acquisition for leadership challenging [19]. Moreover, the dearth of tailored leadership development programs for leaders in the medical context pushes aspiring leaders to utilize current leadership development initiatives that lack a clear focus on addressing the specific challenges faced by healthcare leaders or that help in addressing contextual issues such as navigating interdisciplinary collaborations and fostering an understanding of local legal systems that often influence practice [36].

Furthermore, when considering interpersonal development, the hierarchical nature of healthcare may hinder leadership development. Traditional healthcare structures prioritize individual achievements and performance, this may discourage potential relational leaders from pursuing leadership roles [37,38]. Another pertinent challenge is leadership succession planning [3]. Many institutions fail to identify and foster leadership, leading to potential disruptions in the continuity of leadership within institutions. The lack of diversity and inclusion of underrepresented groups, such as women in leadership positions is a significant issue. This limitation hinders addressing emerging health disparities effectively [36,37].

Healthcare faces several challenges. Such challenges include limited access to quality healthcare services, especially in remote areas, resulting in disparities in healthcare services. Moreover, there is a need to re-skill and up-skill the healthcare workforce to meet emerging healthcare demands [31,39]. In addition, there is a great emphasis on emerging technologies and how they are to be utilized to enhance healthcare services. Such technologies include artificial intelligence, telemedicine, electronic health records, and robotics and automation. Finally, healthcare financing and insurance systems are currently taking shape in many parts of the globe and are fast becoming essential skills for leaders within healthcare.

To address healthcare challenges successfully, a complete understanding of the intricate and ever-changing components of medical leadership development is required. Effective leadership at various levels of an organization requires certain attributes such as self-awareness, problem-solving talents, and social intelligence. This is especially significant given the complexity of the healthcare system [21]. Healthcare necessitates the continued development of business- and strategy-focused competencies in order to advance to higher-level roles. Furthermore, a leader's skills will have a significant impact on how successfully they perform in the future. This stresses the significance of career advancement and specialized professional development programs for healthcare executives. These types of training are critical in equipping physicians with the skills and knowledge they need to deal with the rising healthcare concerns [40,41].

The prevalent patterns in academic studies on leadership development stress the importance of having a multi-level perspective. This entails rigorously scrutinizing how leaders emerge and exercise leadership, as well as how they interact with others and the greater business culture [37]. The emphasis on positive attributes such as trust and respect for one another emphasizes the importance of social capital in developing effective working relationships.

Implications for policy, practice, and research

In terms of policy, there is a need for ones that cater to the recruitment and development of leadership. Policies that target these two areas promote better working environments in addition to healthier and more effective organizations [32]. The continuity of leadership within the confines of medical institutions is in danger if such institutions fail to identify, recruit, groom, and develop potential leaders. Moreover, setting policies that are based on sound needs assessment studies and that promote diversity and inclusion can create a bigger pool to select potential leaders. While previous research has highlighted these policy recommendations, many healthcare organizations still face challenges in recruitment and development. Given the complex systems where healthcare takes place, it is important to recruit those who have the highest tolerance for ambiguity and can navigate the evolving healthcare sector. Moreover, training for such skills requires embracing leadership programs that are longitudinal in nature and workplace-oriented [3]. Here it becomes necessary to recognize that leader development is closely related to administrative positions. As a result, leader development may be a prerequisite to nomination for positions and a requisite during tenure. 

For practice, there is a need to integrate formal leadership training in medical education. It is vital that potential leaders are not discouraged from seeking out leadership roles, as is commonly promoted in traditional academic and healthcare structures [31]. Customized programs in leadership development not only incentivize potential and current leaders but are also crucial measures to elevate leadership competencies within healthcare [39]. Integrated programs relieve potential and current leaders from the burden of seeking out programs that may or may not be suitable for their current roles. Institutions clearly identifying the target programs that potential leaders enroll in help keep focus on the skills and knowledge that are needed for their context [36].

For research, further studies should aim to explore the juxtaposition of leader development with identity development. For example, how much of a leader’s identity is developed during each stage of their leadership journey. Do a group of leaders have a shared leadership identity given that they all belong to the same medical institution? This is important against the backdrop of interprofessional practice, that is, how shared leadership identity is nurtured not only amongst a group of physicians but also amongst a multi-professional healthcare team. Research in the area of leader and leadership development can identify and highlight key areas of interest within the field. Furthermore, researchers should evaluate leadership development in terms of process, starting from identifying who is fit to lead to how to further develop a seasoned leader to developing a group of leaders who are in different stages of their leadership journey. This line of thinking impacts medical education, as leader and leadership development must be integrated at an early stage, beginning in undergraduate programs and throughout postgraduate programs and across different specialties. Such training measures cannot be considered impactful unless impact is truly measured. Hence, research focusing on how to measure the impact of leader and leadership development within healthcare is quite vital in providing insight into the effectiveness of such programs.

Limitations

First, given the method's limitations, a wider search strategy, including more databases and ensuring cross-disciplinary data collection, may have yielded richer results. Second, the review was written by one person. A multidisciplinary team, including a leadership expert and other health professionals, might have provided richer insights and strengthened the arguments put forth.

## Conclusions

This paper discusses the advancement of leaders and leadership development in the medical field with a special emphasis on comprehending the individual and community components. Moreover, it focuses on concerns and repercussions relating to recruitment and development of leadership policies, practices, and planned research projects. Combining these two aspects of leadership development provides a comprehensive perspective that is required for successfully navigating the complex healthcare environment. It further explores the leader and leadership challenges and implications for policy, practice, and research. As an active area of study, and given the ever-evolving nature of the healthcare sector, much interest has been shown to equip leaders with the tools necessary for effective leadership. Facing challenges, such as the emergence of diseases, disease complications, and technological advances, leaders are constantly put in situations that require new ways to approach challenges, thinking-on-your-feet problem-solving skills, and a high tolerance threshold for ambiguities within the organization.

Given the wide-held belief that conscious education and development can increase leadership competencies, resources have been allotted in medical and academic programs in this vein. As such, opportunities and initiatives that cater to developing leaders and leadership have been a focus in the healthcare context. Some approaches used in leadership development programs are short courses, mentoring, and shadowing opportunities. Leader development and leadership development are not one and the same. Leader development focuses on the individual and their identity and competencies to lead while leadership development is more rooted in the process of developing more than one leader and harnessing the interpersonal relationships that ultimately one draws on to accomplish organizational goals.

## References

[1] Steinert Y, Naismith L, Mann K (2012). Faculty development initiatives designed to promote leadership in medical education. A BEME systematic review: BEME Guide No. 19. Med Teach.

[2] Frich JC, Brewster AL, Cherlin EJ, Bradley EH (2015). Leadership development programs for physicians: a systematic review. J Gen Intern Med.

[3] McCauley CD, Palus CJ (2021). Developing the theory and practice of leadership development: a relational view. Leadersh Q.

[4] Kumar B, Swee ML, Suneja M (2020). Leadership training programs in graduate medical education: a systematic review. BMC Med Educ.

[5] Day DV, Fleenor JW, Atwater LE, Sturm RE, McKee RA (2014). Advances in leader and leadership development: a review of 25 years of research and theory. Leadersh Q.

[6] Tedla BA, Hamid AS (2022). Leadership in healthcare organizations. Int J of Health Sci.

[7] Sze GW, Yuin YS, Durganaudu H, Pillai N, Yap CG, Jahan NK (2021). Narrative review of leadership development programs among medical professionals. Open Access Library Journal.

[8] van Diggele C, Burgess A, Roberts C, Mellis C (2020). Leadership in healthcare education. BMC Med Educ.

[9] Samuel A, Durning SJ (2021). Enhancing leadership training through an experiential approach: an online model for the 21st century. Adult Learning.

[10] Watkin A (2019). The leadership laboratory: a leadership practice field involving peer mentors in a first-year, project-based class. Journal of Leadership Education.

[11] Day DV, Sin HP (2011). Longitudinal tests of an integrative model of leader development: charting and understanding developmental trajectories. The Leadership Quarterly.

[12] Day DV (2001). Leadership development: a review in context. The Leadership Quarterly.

[13] Day DV, Dragoni L (2015). Leadership development: an outcome-oriented review based on time and levels of analyses. Annu Rev Organ Psychol Organ Behav.

[14] James E, Evans M, Mi M (2021). Leadership training and undergraduate medical education: a scoping review. Med Sci Educ.

[15] Day DV, Harrison MM, Halpin SM (2009). An integrative theory of leadership development: connecting adult development, identity, and expertise.

[16] Lyons O, George R, Galante JR, Mafi A, Fordwoh T, Frich J, Geerts JM (2021). Evidence-based medical leadership development: a systematic review. BMJ Lead.

[17] Till A, McKimm J, Swanwick T (2020). The importance of leadership development in medical curricula: a UK perspective (stars are aligning). J Healthc Leadersh.

[18] Turner P (2018). Leadership development practices. Leadersh Healthc.

[19] Law AD, Rodin D, Giuliani M, Moraes FY (2020). Championing leadership development in healthcare. Nat Biotechnol.

[20] Cohn S, Clinch M, Bunn C, Stronge P (2013). Entangled complexity: why complex interventions are just not complicated enough. J Health Serv Res Policy.

[21] Sturmberg JP (2016). “Returning to holism”: an imperative for the twenty-first century. The value of systems and complexity sciences for healthcare.

[22] Plsek PE, Wilson T (2001). Complexity, leadership, and management in healthcare organisations. BMJ.

[23] Baker GR, Denis JL (2011). Medical leadership in health care systems: from professional authority to organizational leadership. Public Money & Management.

[24] Hill F, Stephens C (2005). Building leadership capacity in medical education: developing the potential of course coordinators. Med Teach.

[25] Morgeson FP, DeRue DS, Karan EP (2010). Leadership in teams: a functional approach to understanding leadership structures and processes. J Manag.

[26] Alwazzan L, Al-Angari SS (2020). Women's leadership in academic medicine: a systematic review of extent, condition and interventions. BMJ Open.

[27] Alilyyani B, Kerr MS, Wong C, Wazqar DY (2022). An integrative review of nursing leadership in Saudi Arabia. Nurs Open.

[28] Komives SR, Longerbeam SD, Owen JE, Mainella FC, Osteen L (2006). A leadership identity development model: Applications from a grounded theory. J Coll Stud Dev.

[29] Khumalo N, Dumont KB, Waldzus S. (2022). Leaders’ influence on collective action: an identity leadership perspective. Leadersh Q.

[30] Reichard RJ, Johnson SK (2011). Leader self-development as organizational strategy. Leadersh Q.

[31] Marshall-Mies JC, Fleishman EA, Martin JA, Zaccaro SJ, Baughman WA, McGee ML (2000). Development and evaluation of cognitive and metacognitive measures for predicting leadership potential. Leadersh Q.

[32] Mumford TV, Campion MA, Morgeson FP (2007). The leadership skills strataplex: leadership skill requirements across organizational levels. Leadersh Q.

[33] Cullen-Lester KL, Maupin CK, Carter DR (2017). Incorporating social networks into leadership development: a conceptual model and evaluation of research and practice. Leadersh Q.

[34] Galli EB, Müller-Stewens G (2012). How to build social capital with leadership development: lessons from an explorative case study of a multi-business firm. Leadersh Q.

[35] Gentry WA, Martineau JW (2010). Hierarchical linear modeling as an example for measuring change over time in a leadership development evaluation context. Leadersh Q.

[36] Ladegard G, Gjerde S (2014). Leadership coaching, leader role-efficacy, and trust in subordinates. A mixed methods study assessing leadership coaching as a leadership development tool.. Leadersh Q.

[37] Servey JT, Hartzell JD, McFate T (2020). A faculty development model for academic leadership education across a health care organization. J Med Educ Curric Dev.

[38] Avolio BJ, Gardner Gardner, WL WL (2005). Authentic leadership development: getting to the root of positive forms of leadership. Leadersh Q.

[39] Saha JB (2020). Leadership in health care. J Compr Health.

[40] Jamali MC, Purohit H, Tanwar V, Abushohada MAM (2022). Comparative study of healthcare management and leadership skills of health management systems. Int J Health Sci.

[41] Nzinga J, Boga M, Kagwanja N (2021). An innovative leadership development initiative to support building everyday resilience in health systems. Health Policy Plan.

